# Beyond Body Weight-Loss: Dietary Strategies Targeting Intrahepatic Fat in NAFLD

**DOI:** 10.3390/nu12051316

**Published:** 2020-05-06

**Authors:** Nicolai Worm

**Affiliations:** Department of Nutrition, German University for Prevention and Health Care Management, 66123 Saarbrücken, Germany; n-worm@dhfpg-bsa.de

**Keywords:** fatty liver, NAFLD, de novo lipogenesis, weight loss, nutritional therapy meal replacement, low-carbohydrate diet, Mediterranean diet

## Abstract

Non-alcoholic fatty liver disease (NAFLD) has emerged as the most prevalent liver disease in industrialized countries. It is regarded as the hepatic manifestation of the metabolic syndrome (MetS) resulting from insulin resistance. Moreover, insulin resistance impairs glycogen synthesis, postprandially diverting a substantial amount of carbohydrates to the liver and storing them there as fat. NAFLD has far-reaching metabolic consequences involving glucose and lipoprotein metabolism disorders and risk of cardiovascular disease, the leading cause of death worldwide. No pharmaceutical options are currently approved for the treatment of NAFLD. Exercise training and dietary interventions remain the cornerstone of NAFLD treatment. Current international guidelines state that the primary goal of nutritional therapy is to reduce energy intake to achieve a 7%–10% reduction in body weight. Meal replacement therapy (formula diets) results in more pronounced weight loss compared to conventional calorie-restricted diets. However, studies have shown that body mass index (BMI) or weight reduction is not obligatory for decreasing hepatic fat content or to restore normal liver function. Recent studies have achieved significant reductions in liver fat with eucaloric diets and without weight loss through macronutrient modifications. Based on this evidence, an integrative nutritional therapeutic concept was formulated that combines the most effective nutrition approaches termed “liver-fasting.” It involves the temporary use of a low calorie diet (total meal replacement with a specific high-protein, high-soluble fiber, lower-carbohydrate formula), followed by stepwise food reintroduction that implements a Mediterranean style low-carb diet as basic nutrition.

## 1. Introduction

A non-alcoholic fatty liver is defined as one with hepatic steatosis in the absence of significant alcohol consumption or other causes of secondary steatosis [1]. The most commonly used acronym is NAFLD (non-alcoholic fatty liver disease). NAFLD encompasses the entire spectrum of liver diseases characterized by increased fat storage in the liver from steatosis without inflammation (non-alcoholic fatty liver; NAFL) to non-alcoholic steatohepatitis (NASH), cirrhosis, liver failure, and/or hepatocellular carcinoma [1,2]. Though the risk of NAFLD increases with increasing body mass index (BMI) and increasing age [3], it is not uncommon to occur in people with a lean BMI [4].

NAFLD is often referred to as the liver component of a metabolic syndrome. It was not until recently that its relationship to metabolic-vascular disorders such as dysglycemia, dyslipidemia, inflammation, oxidative stress, coagulation disorders with pro-coagulant states, and arterial hypertension was recognized [5]. NAFLD itself is now seen as an independent risk factor for cardiovascular disease [6] that significantly increases the risk of chronic kidney disease and elevates the risk of type 2 diabetes (T2DM) [7,8,9]. In patients with T2DM, the co-existence of NAFLD doubles cardiovascular disease risk [10].

In Western countries, the prevalence of NAFLD ranges from 30% to 40% in the general population [1], whilst risk increases to greater than twofold in patients with T2DM, where it is reported in 60%–70% of these patients [11]. The latest figures for Germany (diagnosed by magnetic resonance imaging) stem from the SHIP (Study of Health in Pomerania) study in the German State “Mecklenburg-Vorpommern;” here, the NAFLD prevalence was 42% among participants aged 42–62 [12]. Given the rising obesity-related health problems and trends toward an aging population, the prevalence of NAFLD is projected to increase nationally and globally [3].

As NAFLD has far-reaching metabolic consequences, must be viewed as a mayor threat to public health [13], and no pharmaceutical options are currently approved for its treatment, lifestyle interventions (exercise training and dietary intervention with changes in quality and/or quantity) remain the cornerstone of NAFLD treatment [1]. This review focuses solely on various dietary concepts.

## 2. Nutritional Aspects of Pathogenesis

NAFLD has long been viewed as a condition that develops due to a chronically positive energy balance as a result of a high-calorie diet and a sedentary lifestyle. Accordingly, an excess of energy alone would cause an increased storage of lipids in both the adipose tissue and the liver. However, NAFLD also occurs in 10%–20% of normal weight Europeans and Americans [4].

More precisely, hepatic steatosis (with relevant genetic predisposition) is the result of an imbalance between fat input and fat output in the liver [14,15,16]. Fat output from liver tissue includes fat oxidation and the secretion of triglyceride-rich, very low-density lipoproteins [16]. According to a study in which patients were infused with and orally fed stable isotopes for four days in order to label and track serum non-esterified fatty acids before having their liver biopsied [14], fat input occurs from three sources: Most liver fat (59%) originates from the lipolysis of adipocytes. The second largest contribution to liver fat in NAFLD patients comes from carbohydrate input via de novo lipogenesis (DNL) with (26%), and the third largest proportion comes from dietary fat (15%).

If the storage function of the adipocytes is impaired, lipid overflow and alternative storage in the form of ectopic fat deposits occur. Ectopic fat is deposited as visceral fat and in organs such as the liver, the pancreas, the heart, the area around the heart, the kidneys, and skeletal muscle [12,17].

### 2.1. Hyperinsulinemia Triggers “De Novo Lipogenesis” from Carbohydrates

Increased de novo lipogenesis (DNL) is pathognomonic for NAFLD and stems from insulin resistance and/or compensatory hyperinsulinemia [18,19]. Postprandially increased blood glucose combined with a high insulin concentrations activates two transcription factors—sterol regulatory element-binding protein 1c (SREBP-1c) and carbohydrate-responsive element binding protein (ChREBP)—in fat cells and liver cells, yielding greater expression of various lipogenic enzymes involved in DNL [20]. This produces a pool of saturated fatty acids, mainly with 16 and 18 carbon atoms: palmitic acid (16:0) and stearic acid (18:0). Their storage in these tissues is associated with a higher risk of metabolic disturbance [21,22]. The negative consequences of insulin resistance and/or hyperinsulinemia are further exacerbated by the fact that NAFLD decreases hepatic insulin clearance, impairs glucose disposal, and increases hepatic glucose production [23]. 

There is a strong relationship between DNL and carbohydrate intake. Excess carbohydrate intake elevates DNL and hepatic glucose production [24]. In obese individuals fed a eucaloric diet containing the usual carbohydrate intake (45–47 En% or 332–374 g carbohydrates from starch and sugar per day, respectively), three-to-four times higher DNL levels were observed in individuals with hyperinsulinemia compared to those with normal and/or low insulin levels [25]. Furthermore, increased carbohydrate intakes also proportionally affect the DNL in normal weight, insulin-sensitive individuals. When high amounts of carbohydrates were provided (e.g., 67 En% carbohydrates or 440 g/day) to insulin-sensitive, normal weight subjects, a four-to-five times increase in DNL was observed [25]. Further, a carbohydrate-rich eucaloric diet paired with a lower protein content (5% of energy) resulted in a significant increase in DNL among insulin-sensitive, normal-weight people [26]. In addition, despite the normal glucose tolerance enabled by hyperinsulinemia, insulin resistance impairs glycogen synthesis, such that a substantial amount of carbohydrates are postprandially diverted to the liver and stored there as fat, ultimately resulting in hepatic steatosis [27].

Fructose consumption plays a special role. A high intake of isolated fructose and sucrose, especially in the form of fruit juice and sugar-sweetened beverages, has been linked to the promotion of fat storage in the liver [28,29]. However, the data are inconsistent, with the risk being associated with dietary intake and increasing with physical inactivity [29]. 

### 2.2. Dietary Fats Are Involved

Approximately 15% of hepatic triglyceride production originates from dietary fatty acid intake [14]. The consumption of saturated fatty acids is often considered a relevant risk factor for NAFLD. However, its effect on hepatic lipid accumulation is often modulated by the type of diet consumed, mainly in a state of excessive calorie intake. Moreover, the relationships between increased saturated fatty acid consumption and liver fat accumulation in an eucaloric diet have not been not sufficiently investigated, and the available data are inconsistent [30,31,32]. Furthermore, the contributions of individual types of fatty acids on liver fat accumulation are often debated [31,32]. Other structural forms of fatty acids, including monounsaturated and polyunsaturated fatty acids, have been suggested to have protective effects against hepatic steatosis by regulating lipid metabolic pathways and the gene expression of important enzymes [30,33] (see below). From a practical perspective, recommending foods rather than isolated nutrients may be preferable. The consumption of highly unsaturated omega-3 fatty acids from fish and krill oil is associated with decreased NAFLD risk, mainly through their action on increasing fatty acid oxidation while reducing lipogenesis (16). Similarly, the high intake of extra virgin olive oil (EVOO) rich in monounsaturated fatty acids is also associated with reduced steatosis [16]. 

## 3. Nutritional Therapy

As there is no sufficient evidence regarding the testing of nutrition interventions for resolving later stage conditions within the NAFLD spectrum, the scope of this review was limited to steatosis. Thus, the aim of this review was to specifically address dietary interventions that have been linked to the mobilization of intrahepatic and intrapancreatic fat stores.

Current international guidelines state that the primary goal of nutritional therapy is to reduce energy intake by 500–1000 kcal per day to achieve a 7%–10% reduction in body weight [1,34,35]. However, studies have shown that BMI or weight reduction is not obligatory for decreasing hepatic fat content or to restore normal liver function [36] (see below: “Eucaloric diets”). On the contrary, it seems more important to fall below the individual visceral/ectopic adiposity threshold, which causes the dysfunction of the liver and pancreas [37]. For example, significant and clinically relevant reductions in liver fat have been achieved independently of the degree of BMI reduction with a low calorie diet using meal replacements [37], as well as with a low-carbohydrate Mediterranean style diet [38].

The pros and cons of different dietary approaches are discussed in the following paragraphs, resulting in a possibly “ideal” integrative dietary intervention to improve or even resolve NAFLD. 

### 3.1. Hypocaloric Diets

Calorie restriction, regardless of the macronutrient composition, to induce negative energy balance is associated with NAFLD improvement and the resolution of hepatic fat [39,40]. These studies have consistently found the improvement in liver fat to be directly associated with weight loss, thus inducing changes in the body fat storage. However, carbohydrate restriction in the context of a hypocaloric diet has been proven in several studies to have a superior effect in comparison to a fat-reduced approach.

#### 3.1.1. Low-Carbohydrate Dietary Patterns

Within a few weeks of calorie restriction, a low-carb diet significantly reduced liver fat content compared to an equally energy-reduced low-fat diet [41]. At three months with a comparable weight loss in both low carb and low-fat diets, a significantly greater hepatic insulin sensitivity and a lower basal glucose level could be detected in a low carb arm [42]. A study conducted in NAFLD patients demonstrated that a diet solely restricted in carbohydrates (<20 g/day) reported a significantly greater reduction in liver fat when compared side-by-side with a calorie-restricted diet (1200–1500 kca/day) even when similar levels of weight loss were achieved in both the interventions [43]. This study and several other studies suggested that carbohydrate restriction has an independent effect on resolving hepatic lipid accumulation, irrespective of the degree of weight loss [43,44,45]. Furthermore, a recently pooled meta-analysis from 10 studies reported that in patients with NAFLD, low carbohydrate intervention significantly reduces hepatic lipid content [46].

In a recently published 18-month randomized controlled intervention trial that examined the effects of isocaloric Mediterranean low-carb versus low-fat diets in a cohort of overweight and obese subjects, the Mediterranean low-carbohydrate diet resulted in a significantly greater reductions in liver, pancreas, and pericardium fat stores, as well as more pronounced improvements in cardiometabolic risk markers, such as triglycerides [38]. Both dietary interventions resulted in similar amounts of weight loss, and the difference in fat deposit level achieved in both the diets remained significant even after accounting for weight loss [47].

Moreover, a longitudinal study of a ketogenic diet in patients with type 2 diabetes, who have higher risk of developing NAFLD, was recently published [48]. Patients were encouraged to restrict carbohydrate intake to a degree that resulted in a metabolic state referred to as nutritional ketosis (assessed by blood β-hydroxybutyrate concentrations). This digitally-supported ad libitum but carbohydrate-restricted intervention significantly reduced surrogate steatosis and fibrosis scores at one [49] and two years [50]. Following one year of treatment, the proportion of individuals with steatosis was reduced by 20%, with significant improvements in the proportion of individuals without any indication of fibrosis (from 18% at baseline to 33%). The improvements observed in the carbohydrate-restricted group were not observed among the patients receiving usual care with standard dietary recommendations within a traditional diabetes care model.

#### 3.1.2. Meal Replacement Therapy

A significant weight reduction can be rapidly achieved with very low-calorie diets (VLCD) 400–800 kcal/day) and low-calorie diets (LCD) at 800–1200 kcal/day using formula-based meals as part of a meal replacement concept. The desired weight reduction can be achieved in a much shorter time interval (66% faster) than with conventional calorie-reduced diets [51]. Formula diets result in more pronounced weight loss compared to conventional calorie-restricted diets [52,53,54,55,56,57], and one study observed significantly better long-term weight loss maintenance among those who initially lost weight using formula diets [51]. Well-formulated meal replacement diets are nutritionally complete with excellent safety records if initiated under medical supervision and if medications are stopped before beginning [58]. The phase with total diet replacement (with no normal meals, foods or drinks) for 8–12 weeks is intended at supporting the change in mindset, helping abstinence from the temptations of conventional foods and eating habits. This seems to be a key factor for adherence and successful substantial weight loss [58]. Contrary to common belief, the drop-out rate for meal replacement diets has been seen to be lower than in traditional calorie-restricted diets [54,59,60].

The German guidelines for the treatment of obesity endorse the use of formula diets, i.e., the use of total meal replacements supplying 800–1200 kcal/day for persons with a BMI of 30 kg/m^2^ or more for a maximum of 12 weeks [61]. However, not everybody succeeds in losing weight in the long run.

### 3.2. Eucaloric Diets

In the national and international guidelines for NAFLD therapy, the use of modified eucaloric diets have so far been disregarded. In a state of a balanced energy intake without any weight or BMI reduction, the relevance of carbohydrate restriction is worth considering as a possible approach to reduce liver fat. In carbohydrate-restricted diets, starch and sugar are partially replaced by fat, protein, or a combination of both.

#### 3.2.1. The Role of Dietary Fat

Isocaloric Mediterranean diets increasing fat intake to around 40 En% (mainly from adding extra virgin olive oil (EVOO)). EVOO helps in protecting against developing NAFLD and significantly reducing liver fat content [62]. As illustrated in animal studies, the beneficial effect of the Mediterranean diet is believed to be due, at least in part, to its highly enriched EVOO content. EVOO significantly reduces liver fat and helps in resolving the pro-inflammatory state [63,64]. EVOO is concentrated with polyphenols, including hydroxytyrosol. Hydroxytyrosol rescues insulin receptor signaling by mitigating endoplasmic reticulum (ER) stress in adipose and liver tissue. Additionally, EVOO alleviates ultrastructural damage and lipid deposit in the liver while suppressing the hepatic expression of the genes involved in lipogenesis [64].

In addition, the n-3 long-chain polyunsaturated fatty acids eicosapentaenoic acid (EPA; C20:5n-3) and docosahexaenoic acid (DHA; C22:5n-3) exert antisteatotic and antioxidant responses that can prevent or resolve the inflammation or the liver [65]. There have been ample studies suggesting that supplementing long-chain omega-3 fatty acids (EPA and DHA) in the range of 2–5 g per day independently contributes to reductions in liver fat content, a finding that was confirmed by a recent meta-analysis of randomized controlled trials [66]. The beneficial effect of omega-3 fatty acid supplementation has been reported to be independent of weight loss or other dietary manipulations [67]. With regard to long-chain omega 3 fatty acids, there are four biologically plausible mechanisms of action for the effect of EPA and DHA on liver fat content [68]: (a) They influence the PPAR (peroxisome proliferator-activated receptors) system, especially PPARα, and thereby promote fatty acid oxidation in the liver; (b) they suppress the expression of two lipogenic transcription factors SREBP-1C and ChREBP, which are involved in the de-novo lipogenesis of carbohydrates in hyperinsulinemic states; (c) they promote the release of bile acids and thus contribute to the release of fat and cholesterol from the liver; and (d) their increased incorporation into adipose tissue mediates adiponectin production and reduces the susceptibility of the adipocytes to elevated inflammation and contributes to their functional integrity.

#### 3.2.2. The Role of Dietary Protein

Increased protein intake independently contributes to mobilizing liver fat. This high protein effect may be modulated by branched-chain amino acid (BCAA) [69] and methionine content in the diet [70]. For example, following supplementation with 60 g of whey protein per day (3 × 20 g for four weeks) while on an otherwise ad libitum diet, liver fat content decreased significantly by 21% [71]. A recent six-week randomized controlled study by the German Institute of Human Nutrition (DIfE) in Potsdam using a eucaloric, protein-rich diet (30 En% protein, 30 En% fat, and 40 En% carbohydrates) achieved a significant reduction in liver fat content in patients with T2DM and NAFLD versus their habitual diet [72]. The participants in the study were randomized to two different protein groups; one group received protein sources mainly from plants (e.g., legumes), and the other group predominantly received animal protein (e.g., meat and milk products). Both intervention groups reported a significant decrease in liver fat, with a more substantial decrease observed in the animal protein group (–48% versus –36%) in six weeks. The significant resolutions of liver fat in both these intervention arms were associated with reductions in free fatty acid levels and improvements in lipogenesis markers [73]. 

#### 3.2.3. The Role of Dietary Carbohydrate

The reduction of carbohydrate intake and the modification of carbohydrate quality appear to play important roles in nutrition therapy for NAFLD. A recent study assessed a strictly ketogenic eucaloric diet in 10 obese patients with high liver fat for 14 days under controlled conditions. The carbohydrate intake was reduced to 4%, the fat intake increased to 72%, and the protein intake increased to 24% [36]. The subjects consumed, on average, 3115 kcal per day in an attempt to prevent weight loss and to separate the effects of weight loss from carbohydrate restriction, per se, in outcomes. Despite reported good compliance with the intervention, participants lost 1.8% body weight in two weeks. After two weeks, the liver fat content had decreased in all participants by, on average, 44% with minimal weight loss. The decrease in liver fat was accompanied by a reduction in DNL and an increase in fatty acid oxidation.

### 3.3. Protective Dietary Compounds 

Based on the multifactorial pathogenesis of NAFLD and the associated metabolic-vascular risk, other dietary compounds are also relevant, as they, in part, favorably influence the underlying pathophysiology of fatty liver-induced disorders. In this regard, there is good evidence for the targeted use of vitamin E and probiotics [74]. In particular, the effects of the soluble fiber beta-glucan (from oats) and inulin (from vegetables) systemically target fatty livers. In addition, because of their effects on satiety, these dietary fibers are of great importance for compliance with hypocaloric diets [75]. Experimental studies and observational studies have shown the hepatoprotective effects of choline, l-carnitine, and taurine, which are beyond the scope of the current review and cannot be discussed in detail here (reviewed in [76]). Choline (and its naturally occurring metabolite betaine) is a methyl donor that has been shown to improve liver transaminases in patients with NAFLD and to protect against worsening conditions during the therapy of NAFLD [77]. l-carnitine administration was shown to ameliorate or prevent the liver from insult through the augmentation of hepatic mitochondrial beta-oxidation Recent studies have suggested the role of oxidative pathways as favorable targets for the treatment of NAFLD [78,79]. Taurine treatment suppressed the high-fat diet induced reduction of the enzyme activity of hepatic superoxide dismutase and catalase, as well as the reduction of the hepatic level of reduced glutathione and ATP. In HepG2 cells, taurine suppressed the fatty acid-induced lipid accumulation, the production of reactive oxygen species and levels of thiobarbituric acid-reactive substances (TBARS), and the amelioration of the fatty acid-induced disruption of the mitochondrial membrane potential. These results showed that taurine was effective in alleviating hepatic steatosis by reducing oxidative stress. Taurine may therefore be of therapeutic value in reducing the risks associated with NAFLD [80].

## 4. “Liver-Fasting”—An Integrative Nutrition Intervention Concept

Based on the scientific data, an evidence-based nutritional concept was formulated that combines several of the previously discussed nutrition therapies with positive effects on reducing liver fat; this was termed “liver fasting” [81]. It involves the temporary use of low-calorie meal replacement with a specific high-protein (dairy/whey-protein), high-soluble fiber, and lower-carbohydrate formula (Hepafast^®^), followed by stepped food reintroduction (see below) that implements a Mediterranean style low-carb diet for basic nutrition [81,82]. For long-term weight loss maintenance, the “Flexi-Carb” diet has been proposed; this diet has a very limited carbohydrate intake in baseline, but it can be adjusted to individual muscle activity—allowing for increased carbohydrate intake with increasing physical activity [81]. 

“Liver fasting” comprises four phases:

1. Initial phase (two-to-three weeks): meal replacement (Hepafast^®^) three times a day, supplemented with a vegetable preparation two times a day (max. 200 kcal additional), such that 800–1000 kcal are consumed daily.
2. Reduction phase: (about 8–10 weeks) meal replacement twice a day and one Mediterranean low-carb meal (with a maximum of 600 kcal).
3. Stabilization phase (about four weeks): meal replacement once a day and two Mediterranean low carb meals day (with a maximum of 600 kcal each).
4. Maintenance phase: all meals correspond to an individually carbohydrate-adapted Mediterranean weight maintenance diet.

The meal replacement shake is high in soluble fiber (beta-glucan and inulin). In addition to essential fatty acids, it contains nutrients such as choline, carnitine, and taurine. The powder is mixed three times a day with low-fat milk. During the initial phase at lunchtime and in the evening, a large portion of vegetables should be consumed as soup, salad, or raw food with a dip (max 200 kcal per day).

There are several lines of evidence supporting the use of this “liver-fasting” strategy (phase 1) as an effective initial treatment for steatosis. A prospective 14 day follow-up of 60 patients in a clinical setting (University Hospital Homburg) reported the effectiveness of this strategy in significantly improving liver fat, liver stiffness, liver transaminases, fatty liver index (FLI), fasting blood glucose, haemoglobin A1c, blood lipids, and uric acid [83]. On average, the patients lost approximately 4.6% of their body weight, with the majority of the weight loss consisting of body fat. Particularly noteworthy is the examination of this “liver fasting” concept in a 12-week randomized controlled intervention study at the University of Hohenheim [84]. The control group received an isocaloric diet (about 1000 kcal/day) on natural foods based on the “Low Glycemic and Insulinemic Diet” (LOGI-Diet) [85], while the intervention group followed phases 1 and 2 of the liver-fasting protocol with Hepafast^®^ and a Mediterranean style carbohydrate-reduced diet. Even with a comparable intensity of care delivery and weight reduction, the liver-fasting group reduced liver fat content and blood pressure significantly more than the control group.

## 5. Conclusions

A negative energy balance caused by calorie restriction induces a resolution of hepatic fat and an improvement of NAFLD regardless of the diet’s macronutrient composition. However, carbohydrate restriction in the context of a hypocaloric diet has been proven to have a superior effect in comparison to a fat-reduced approach in several studies. Moreover, eucaloric diets (i.e., in a state of a balanced energy) without any weight or BMI reduction, the relevance of carbohydrate restriction (with a proportionately higher intake of protein and/or unsaturated fatty acids) are effective in lowering liver fat content in ways that are significant and clinically meaningful. 

Therefore, an initial (very) low calorie diet period of several weeks on a total diet replacement with a nutritionally complete, high protein, and low-energy formula, followed by a structured program of food reintroduction that implements a Mediterranean style low-carbohydrate diet may be viewed as an optimal nutritional therapy for patients with NAFLD (and type-2 diabetes).
